# Mutual interplay between cognitive offloading and secondary task performance

**DOI:** 10.3758/s13423-023-02312-3

**Published:** 2023-06-13

**Authors:** Sandra Grinschgl, Frank Papenmeier, Hauke S. Meyerhoff

**Affiliations:** 1 Department of Psychology, University of Graz, Universitätsplatz 2, 8010 Graz, Austria; 2 Department of Psychology, University of Tübingen, Tübingen, Germany; 3 University of Erfurt, Erfurt, Germany; 4 Leibniz-Institut für Wissensmedien, Tübingen, Germany

**Keywords:** Cognitive offloading, Working memory, Secondary task performance, Dual-task

## Abstract

Various modern tools, such as smartphones, allow for cognitive offloading (i.e., the externalization of cognitive processes). In this study, we examined the use and consequences of cognitive offloading in demanding situations in which people perform multiple tasks concurrently—mimicking the requirements of daily life. In a preregistered study, we adapted the dual-task paradigm so that one of the tasks allowed for cognitive offloading. As a primary task, our participants (*N* = 172) performed the pattern copy task—a highly demanding working memory task that allows for offloading at various degrees. In this task, we manipulated the temporal costs of offloading. Concurrently, half of the participants responded to a secondary *N*-back task. As our main research question, we investigated the impact of offloading behavior on secondary task performance. We observed that more pronounced offloading in the condition without temporal costs was accompanied by a more accurate performance in the *N*-back task. Furthermore, the necessity to respond to the *N*-back task increased offloading behavior. These results suggest an interplay between offloading and secondary task performance: in demanding situations, individuals increasingly use cognitive offloading, which releases internal resources that can then be devoted to improving performance in other, concurrent tasks.

**Supplementary Information:**

The online version contains supplementary material available at 10.3758/s13423-023-02312-3.

Individuals perform various cognitive tasks daily, such as reading, counting, or memorizing important information. One way to reduce internal cognitive demands emerging from such tasks is to offload (i.e., externalize) cognitive processes into the environment. Particularly, modern tools, such as smartphones or tablets, are practical external aids; and most people frequently use them for storing important information (e.g., storing appointments in a calendar or writing a shopping list) rather than internal memorization (Finley et al., 2018). While cognitive offloading lowers cognitive demands in the task at hand (Risko & Gilbert, 2016), it can also support the memorization of later information (Storm & Stone, 2015) and subsequent task performance (Runge et al., 2019). Nevertheless, it is unclear if (and how) cognitive offloading can be used beneficially *during* the performance of highly demanding tasks beyond a potential reduction of cognitive demands (i.e., beyond pure externalizations).

In theory, cognitive offloading is supposed to release cognitive resources that might be devoted to other cognitive tasks (Kirsh, 2010; O’Hara & Payne, 1998). However, an empirical demonstration of such an immediate release and redirection of cognitive resources is still lacking. Thus, as our main research endeavor, we aimed at testing whether offloading in one task releases cognitive resources that might support the immediate performance in another concurrent but unrelated task. Doing so, we investigate the mutual interplay between offloading memory processes in one task and the concurrent performance of a secondary task using an adapted dual-task paradigm that allows for offloading in the primary task.^1^

Cognitive offloading—defined as physically interacting with one’s environment to externalize cognitive processes (Risko & Gilbert, 2016)—enables individuals to overcome the limitations of the human cognitive architecture, such as the limited working memory capacity (Baddeley, 2003; Luck & Vogel, 2013). For instance, offloading working memory increases the amount of information that can be maintained (Risko & Gilbert, 2016; Wilson, 2002). Another benefit of cognitive offloading for working memory might be the release of cognitive resources.^2^ The *flexible resource model* of working memory suggests that working memory entails a central pool of resources that can be flexibly distributed between competing stimuli (e.g., Huang, 2010; Luck & Vogel, 2013). Thereby, offloading might be used to free up these resources and to redirect them to the processing of other stimuli. Following the flexible resource model, first studies indicate that offloading indeed releases resources that can be used for long-term memory formation (Grinschgl et al., 2021), storing subsequent information (Storm & Stone, 2015), or processing subsequent tasks (Runge et al., 2019).

With regard to subsequently performed tasks, previous research has documented beneficial effects of cognitive offloading in the first task on performance in the following task. When Storm and Stone (2015) allowed their participants to offload a to-be-memorized wordlist (i.e., store it in a computer file), they were more accurate at recalling another wordlist than when offloading was prohibited. Runge et al. (2019) replicated and extended this finding to unrelated subsequent tasks. When the participants were allowed to offload a wordlist, they performed better in an unrelated, subsequent arithmetic task. These findings suggest that cognitive offloading can release cognitive resources (e.g., from maintaining information in working memory), which can be used to improve performance in subsequent tasks. However, according to the flexible resource model, it should be possible to immediately redistribute cognitive resources when performing working memory tasks (e.g., Luck & Vogel, 2013) and not only in a subsequent manner.

In daily life, individuals often have to perform multiple tasks at the same time, but empirically it is unclear how cognitive offloading and potentially released resources interact with performance in these highly demanding situations. Performing tasks in dual- and multitask setups usually poses more interference between the tasks than when they are performed sequentially (Nijboer et al., 2013; Strobach & Schubert, 2017). Therefore, particular situations with concurrent tasks raise the question how this interference can be compensated by offloading. Importantly, this question is not trivial as the effort of offloading behavior (e.g., increased physical interaction with a device) might draw upon the limited cognitive resources when they are already under pressure. In such a scenario, cognitive offloading might not act beneficially on performance in dual-task settings as it might raise extraneous load (i.e., task-irrelevant load) in addition to an already high intrinsic load (i.e., task-relevant load; see Paas et al., 2003). In contrast, the generally lower cognitive load in sequential task settings might not hinder the beneficial effects of offloading on subsequent task performance (see Runge et al., 2019).

Some studies showed that offloading information within a primary task can be beneficial to process additional information within a highly related secondary task using the same stimuli (e.g., Chen et al., 2017; Gilbert, 2015). However, a systematic study of the interplay of offloading in dual-tasks with a high cognitive load would be necessary to promote theorizing on the interplay between task load and offloading behavior. Particularly, it is unknown whether the released resources that emerge from offloading behavior in a primary task could be used to improve performance in another concurrent, but unrelated task. Based on the flexible resource model, the distribution of cognitive resources might even be possible across different kinds of stimuli (e.g., visual and auditory stimuli; see Ma et al., 2014). Thus, more offloading in a visual primary task might foster performance in an auditory secondary task due to transferring cognitive resources to the secondary task–which would be a so far unexplored benefit of cognitive offloading. Alternatively, offloading might not release cognitive resources in highly demanding situations. This could be due to additional load for performing the offloading operation or due to an inability of transferring the released resources to a very dissimilar secondary task (i.e., to the processing of unrelated stimuli). This latter assumption would support a competing working memory theory, namely the *discrete slot model.*^3^ This model suggests that working memory resources are separated by slots without sharing resources between slots (e.g., see Huang, 2010; Souza et al., 2014; for empirical support see Barton et al., 2009).^4^ Similarly, the *multiple-component working memory model* suggests that visual and auditory information is stored in separate storage systems of working memory (e.g., Baddeley, 2003; see Cocchini et al., 2002, for empirical support). Thus, based on these theories the release of cognitive resources due to offloading in a visual task might not benefit performance in a current auditory task as resources cannot be divided between those tasks.

A novel investigation of cognitive offloading in demanding situations is required to achieve a greater theoretical understanding of the underlying interplay of cognitive resources. Thereby, the empirical support for different working memory models in the context of cognitive offloading can be tested. More specifically, such an investigation could extend the classical working memory theories—especially the flexible resource model—to the use of cognitive offloading as a strategy for freeing up working memory resources. Furthermore, it is also of great practical relevance. Performing multiple tasks concurrently is highly prevalent in everyday life (Colom et al., 2010) and work settings (Appelbaum et al., 2008; Kirchberg et al., 2016). Unsurprisingly, performance often decreases when multiple tasks are performed concurrently rather than in isolation (e.g., Cowan & Morey, 2007; Heathcote et al., 2015; Saults & Cowan, 2007). However, a higher working memory capacity (i.e., more available resources) is associated with a faster or more accurate multitasking performance (Colom et al., 2010). Hence, using cognitive offloading to release working memory resources might be a useful strategy for improving performance when engaging in multiple tasks in daily life and work settings.

To investigate the mutual interplay between cognitive offloading and secondary task performance, we used a classical dual-task paradigm with an important adaption, namely, allowing for offloading in the primary task. Our participants performed the pattern copy task (PCT) during which they copied a color pattern from a model window into a workspace window, whereby only one of the windows was visible at a time. Therefore, this task reflects a load-intensive working memory task.^5^ Given the demands of the task, it cannot be solved without switching between the model and workspace windows. These switches reflect cognitive offloading in our paradigm (i.e., the physical effort that is invested to reduce demands; e.g., Fu & Gray, 2000; Gray et al., 2006) and might vary depending on the experimental conditions. We manipulated the temporal costs for accessing the model window—half of the participants could open it without any costs, whereas the remaining half experienced a 2-s lockout each time accessing it. In previous studies, within a single-task setup, this manipulation of temporal costs has reduced offloading behavior (e.g., see Grinschgl et al., 2020). Orthogonally to the cost manipulation, we asked half of our participants to perform an auditory *N*-back task, which is a classical working memory task that (in our study) did not allow for offloading (Jaeggi, Buschkuehl, et al., 2010a; Jaeggi, Studer-Luethi, 2010b). The remaining half of the participants ignored the stimuli from the *N*-back task.

As our main research question, we aimed at testing whether offloading releases cognitive resources that can be used to foster secondary task performance. Participants who experienced no temporal costs in the PCT might show a more accurate and/or faster performance in the *N*-back task when compared with participants who experience temporal costs in the PCT. Thus, more offloading in the PCT (due to the absence of temporal costs) should release working memory resources that might be used for performance improvements in the *N*-back task (as suggested by the flexible resource model). As a prerequisite for this main research question, we wanted to test whether temporal costs and demand manipulations that usually impact offloading behavior in single-task studies, also impact offloading in our dual-task study. Replicating previous studies, in the condition ignoring the secondary task we expected more pronounced offloading behavior in the pattern copy task when no temporal costs were associated with offloading than . Furthermore, we tested whether the necessity to respond to the *N*-back task impacts offloading behavior. Due to a higher cognitive demand caused by the necessity to respond to the concurrent secondary task, participants in this condition should offload more than those who do not need to respond to the *N*-back task. Doing so, we tested the main effects of our manipulated factors (temporal costs and necessity to respond to the secondary task) but also an interaction that might signal an over- or underadditivity of the influence of these factors on offloading behavior.

Our experiment is an empirical demonstration of two important points—the impact of the necessity to perform a concurrent secondary task on offloading behavior (i.e., a demand manipulation in a dual-task setting) and the impact of cognitive offloading in one task on concurrent secondary task performance. In particular, the investigation of the latter should yield a better theoretical understanding of the processes involved in cognitive offloading and should provide important insights into the theory that offloading releases cognitive resources. Taken together, this study attempts to further understand offloading behavior *during* highly demanding situations when multiple tasks must be performed concurrently.

## Method

This experiment was preregistered at the Open Science Framework (https://osf.io/89t24). The scripts, raw data, and study materials are available at the Open Science Framework (https://osf.io/79arq/).

### Participants

The final sample consisted of 172 participants (130 female, 41 male, one diverse; 18–42 years old, *M*_age_ = 24.31 years, *SD*_age_ = 4.31 years). This sample size was motivated by the following power considerations: Although there is no prior research investigating dual-task setups (regarding cognitive offloading in the PCT), dual-task costs typically arise at medium to large effect sizes in related working memory paradigms (e.g., Cowan & Morey, 2007; Saults & Cowan, 2007). Thus, we considered a medium effect size of *f* = .25 as a reasonable assumption for which we aimed at achieving a high statistical power of (1 − *β*) = .90. The corresponding power analysis (powering for the main effects as well as the interaction effect) suggests a minimum sample size of 171 participants (we tested 172 participants to balance group sizes). As preregistered, we replaced four participants due to missing data, one participant due to noncompliance with task instructions, one participant due to a too low working memory capacity in the Corsi blocks task, six participants due to too low performance in the secondary *N*-back task (sensitivity < 0.5),^6^ and four participants with deviations that were too large (±3 *SD*s) from one of the dependent variables of the PCT. The participants received course credits or monetary compensation. The experimental procedure was approved by the ethics committee of the Leibniz-Institut für Wissensmedien. All participants provided informed consent before testing.

### Apparatus

The experiment was conducted on 12.3″ Microsoft Surface Pro tablets (2,736 × 1,824 pixels). The touch functionality served as an input device for the Corsi blocks task and the PCT. The tablets were lying flat on the table at a viewing distance of approximately 36 cm. The auditory stimuli for the secondary task (*N*-back task) were presented via headphones, and a foot pedal served as an input device for this task. All experimental scripts were coded using PsychoPy libraries (Peirce et al., 2019).

### Stimuli and procedure

Initially, each participant completed the Corsi Blocks Task, which served as a control measure for working memory capacity, followed by the PCT. Concurrent with the PCT, half of the participants responded to the *N*-back task, whereas the other half of the participants ignored the *N*-back task. Thereafter, the participants completed the NASA-TLX questionnaire.

#### Corsi blocks task

The Corsi blocks task served for measuring participants’ working memory capacity (adapted from Milner, 1971). In this task, the participants attended to sequences of spatial locations which they then had to recall from their memory. The spatial sequences were presented in a 5 × 5 grid of empty squares (each 2.52 × 2.52 deg) in which randomly selected squares turned yellow in 1-s intervals. Following this sequence (besides a retention interval of 0.3 s), the participants were required to tap the squares in the correct order. The length of the sequence varied adaptively based on the accuracy of the preceding response. Following an accurate recall of a sequence, the number of squares increased by 1, whereas the number of squares decreased by 1 (with a minimum set size of 2) following an incorrect recall. The first sequence started with a length of two squares. The participants performed 30 trials of this task. To calculate working memory capacity, we averaged the set size of the last 10 correctly solved trials.

#### Pattern copy task

**Fig. 1:**
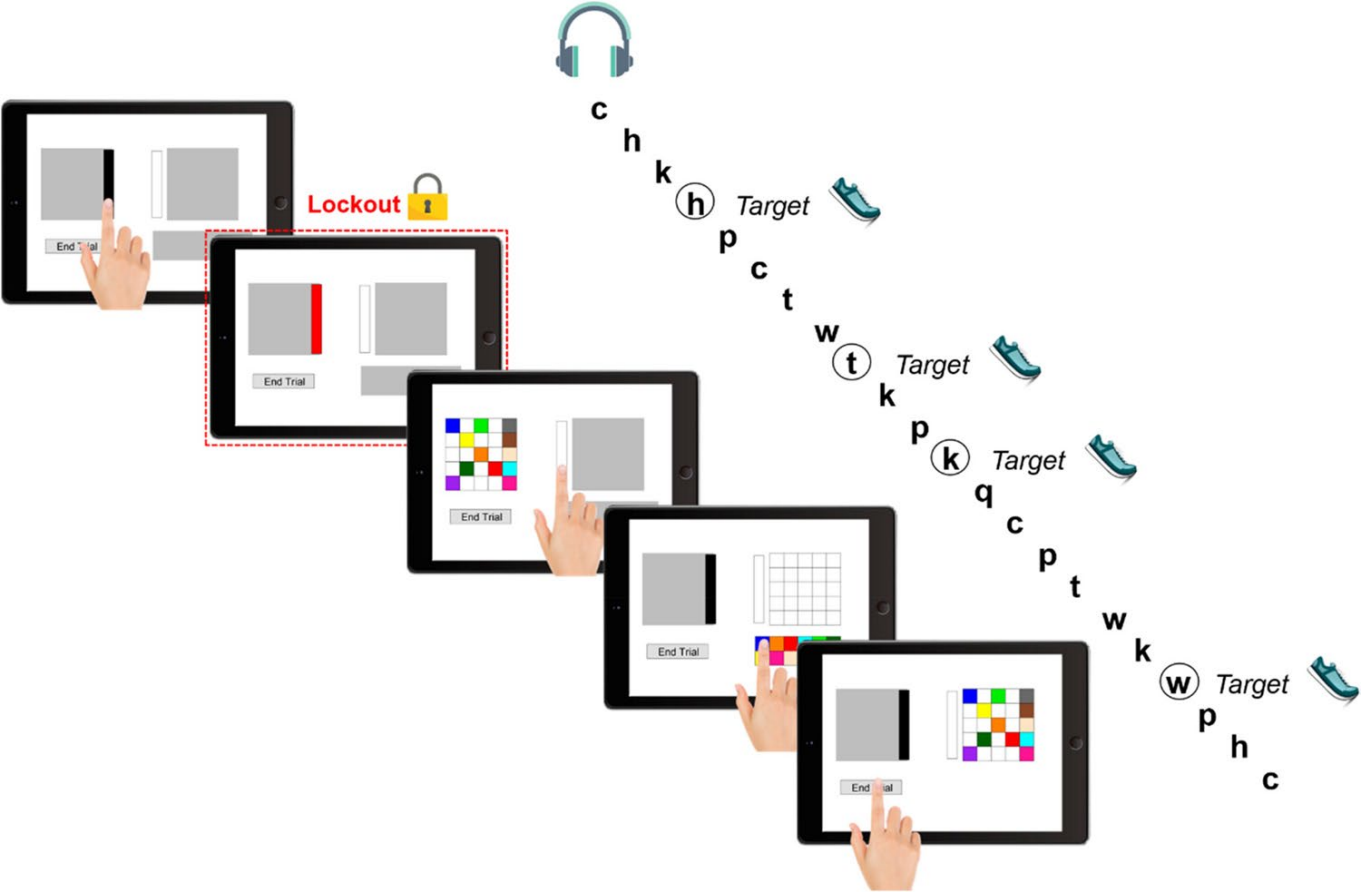
Illustration of the pattern copy task and the *N*-back task. In the PCT, the participants had to copy a pattern of colored squares from a model window (left side) into a workspace window (right side). For half of the participants, there was a 2-s lockout each time they opened the model window (lockout condition), whereas the other half of the participants could open the model window immediately (no lockout condition). In the concurrent *N*-back task, the participants listened to an auditory presentation of consonants in intervals of 3 s. Half of the participants had to respond via a foot pedal when the presented stimulus was the same as the one presented two positions before (respond condition). The other half of the participants ignored the auditory secondary task (ignore condition). The tablet frame was designed by Freepik, the lock and headphones were designed by rawpixel.com/Freepik, and the shoe was designed by macrovector/Freepik. (Color figure online)

The PCT served for measuring working memory offloading (e.g., Fu & Gray, 2000; Gray et al., 2006). The participants were required to copy a pattern of colored squares from a model window (left side of the screen) into a workspace window (right side of the screen, see Fig. 1). The model and workspace window both consisted of a 5 × 5 grid of empty squares (each 2.52° × 2.52°). The grid in the model window was filled with 12 distinct colored squares (blue, orange, red, cyan, green, dark green, yellow, bisque, sienna, purple, pink, and gray). The resource window (located below the workspace window) included the same 12 colored squares arranged in a 2 × 6 grid. The participants dragged and dropped these squares into the workspace window to copy the pattern. Importantly, the participants could either open the model window to inspect the pattern (by clicking on a black bar to its right) or open the workspace and resource window to rebuild the pattern (by clicking on a bar to their left). Whenever the model window was open, the workspace and resource windows were covered by gray masks. Similarly, the model window was covered when the participants accessed the workspace and resource windows. The participants were allowed to switch between the model and workspace/resource windows as frequently as they wanted.

Within this task, the main indicator of cognitive offloading is the number of openings of the model window with more openings indicating more offloading (i.e., additional physical action to reduce the cognitive demand of the individual copy cycles). Between participants, we manipulated the temporal costs of this offloading behavior with a lockout versus no lockout condition. Half of the participants experienced a 2-s lockout each time they accessed the model window. During this time interval, the bar for opening the model window turned red and it needed to be clicked again after the interval to open the window.^7^ The other half of the participants experienced no lockout and were able to access the model window without temporal delays.

The participants were required to copy the pattern correctly before they could undertake the next trial by pressing an “End Trial” button. When the pattern was not copied correctly, a message was displayed asking the participants to keep editing it. The participants completed 20 trials preceded by two practice trials. The patterns of colored squares were generated randomly to the extent that one participant of each experimental condition received the same pattern in the same order (i.e., four participants—one from each group—received the same patterns and order). This was done in order to mitigate a potential impact of the individual patterns on participants’ task performance.

Beyond the number of openings of the model window (our main offloading variable), we also measured offloading behavior with the duration of the first opening of the model window and the number of correctly copied items after the first opening of the model window. The rationale of this is that the first opening—and the subsequent copy cycle—is independent of any previous opening behavior within this trial. For these variables, a shorter duration of the first opening, as well as a lower number of initially (correctly) copied items, indicate increased cognitive offloading and less internally memorized information.

#### *N*-back task

Concurrent to the PCT, we presented an auditory *N*-back task (adapted from Jaeggi et al., 2010a; see Fig. 1). Following the start of each trial of the PCT, we presented a random sequence of consonants (c, g, h, k, p, q, t, and w; German pronunciation) in a male voice.^8^ Every 3 s, a new consonant was presented. The sequence ended when the corresponding trial of the PCT was completed. The task instructions varied between participants. Half of the participants were instructed to ignore the *N*-back task (ignore condition). The remaining participants were instructed to respond to the *N*-back task by pressing the foot pedal as fast and accurately as possible each time the presented consonant matched the one presented two positions before (i.e., 2-back task; respond condition). Thus, to keep our conditions as similar as possible, both the ignore and respond conditions were presented with the *N*-back stimuli but only the respond condition was instructed to react to it.^9^

The consonants were randomized with two restrictions. First, there was an independent probability of .33 for each stimulus to be a target (i.e., a response in the respond condition should be given). Second, no 1-back and 3-back compilations were allowed. To analyze secondary task performance for participants in the respond condition, we excluded the last auditory stimulus of each trial (as participants might not have had the chance to answer to this stimulus) as well as responses with a response time lower than 0.2 s (i.e., anticipations or delayed responses). If multiple responses were recorded for a single stimulus, only the first one was analyzed. As proxies of secondary task performance, we calculated the sensitivity (*d′*) derived from signal detection theory (Macmillan & Creelman, 2004) and the response time for accurate responses averaged across the 20 test trials. A higher sensitivity (*d′*) and shorter response times indicate improved secondary task performance.

#### NASA-TLX

The NASA-TLX is a paper–pencil questionnaire requesting the experienced workload in a preceding task (Hart, 2006; Hart & Staveland, 1988). With this measure, individuals’ perceived workload within and between tasks can be compared (Hart & Staveland, 1988). The participants rate mental demand, physical demand, temporal demand, effort, frustration (each very low to very high), and performance (perfect performance to failure) on a scale from 1 to 21. Thus, the participants rated how mentally and physically demanding they experienced the task(s). With regard to temporal demand, they were asked about the pace at which the individual steps of the task(s) followed each other. Furthermore, they rated how much effort it took them to perform the task(s), how frustrated/annoyed they got during the performance of the task(s), and how successful they judged their performance. Our participants completed a German translation of the NASA-TLX at the end of the experiment. As an index of the overall workload, we averaged each participant’s ratings to those variables (cf. Hart & Staveland, 1988).

## Results

Within this results section, we focus on our main confirmatory and exploratory analyses. Further exploratory analyses can be found in the supplementary materials.

### Cognitive offloading

**Fig. 2:**
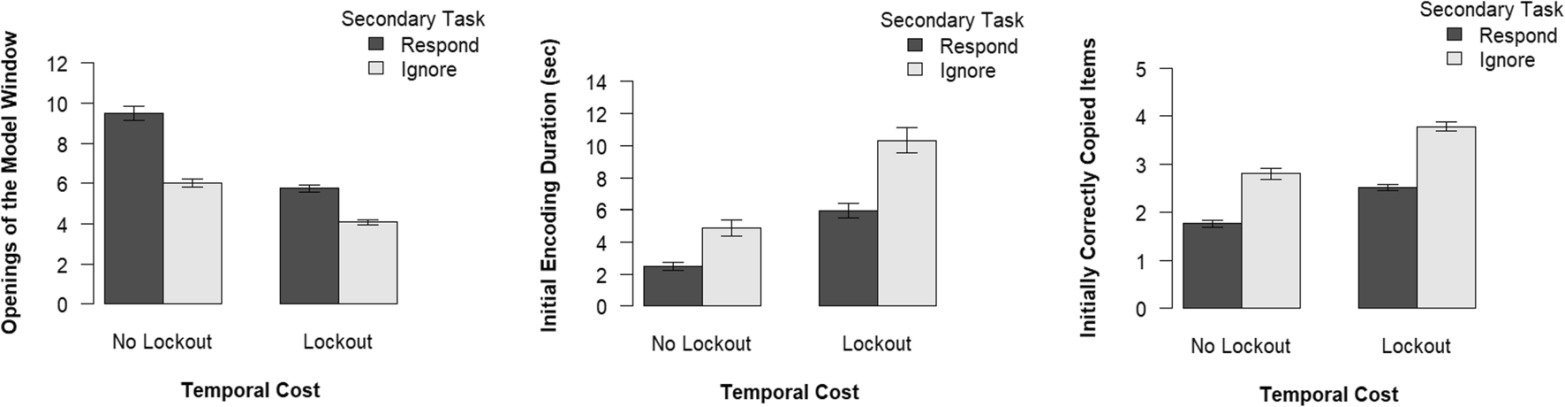
Results of the three measures of offloading behavior separately for each factor combination. Error bars indicate the standard error of the mean

Following our preregistered analysis plan, we conducted separate 2 × 2 between-subjects analyses of variance (ANOVAs) with temporal costs (no lockout vs. lockout) and secondary task conditions (respond vs. ignore) as the independent variables for each of the three dependent variables (i.e., openings of the model window, initial encoding duration, and initially correctly copied items) of the PCT. Participants in the no lockout condition opened the model window more frequently, *F*(1, 168) = 156.15, *p* < .001, η_*p*_^2^ = .48, 95% CI [.37, .56], showed a shorter initial encoding duration, *F*(1, 168) = 68.88, *p* < .001, η_*p*_^2^ = .29, 95% CI [.18, .39], and copied fewer items correctly at the initial opening of the model window, *F*(1, 168) = 87.53, *p* < .001, η_*p*_^2^ = .34, 95% CI [.23, .44], than did participants in the lockout condition. Furthermore, participants responding to the secondary task opened the model window more frequently, *F*(1, 168) = 127.92, *p* < .001, η_*p*_^2^ = .43, 95% CI [.32, .52], showed a shorter initial encoding duration, *F*(1, 168) = 39.91, *p* < .001, η_*p*_^2^ = .14, 95% CI [.09, .29], and copied fewer items initially correctly, *F*(1, 168) = 156.88, *p* < .001, η_*p*_^2^ = .48, 95% CI [.38, .56], than did those who ignored it. For the number of openings of the model window, there was a significant interaction between the temporal costs and secondary task conditions, *F*(1, 168) = 15.92, *p* < .001, η_*p*_^2^ = .09, 95% CI [.02, .17]. As visible in Fig. 2 (left panel), the effect of the secondary task was more pronounced in the no lockout than the lockout condition. Post hoc *t* tests for independent samples confirmed that the secondary task increased offloading behavior in both conditions, all *t*(84)s ≥ 7.97, all *p*s < .001, all η_*p*_^2^s ≥ .43. There were no interactions for the initial encoding duration, *F*(1, 168) = 3.29, *p* = .071, η_*p*_^2^ = .02, 95% CI [.00, .08], and the initially correctly copied items, *F*(1, 168) = 1.74, *p* = .189, η_*p*_^2^ = .01, 95% CI [.00, .06].

### Secondary task performance

**Fig. 3:**
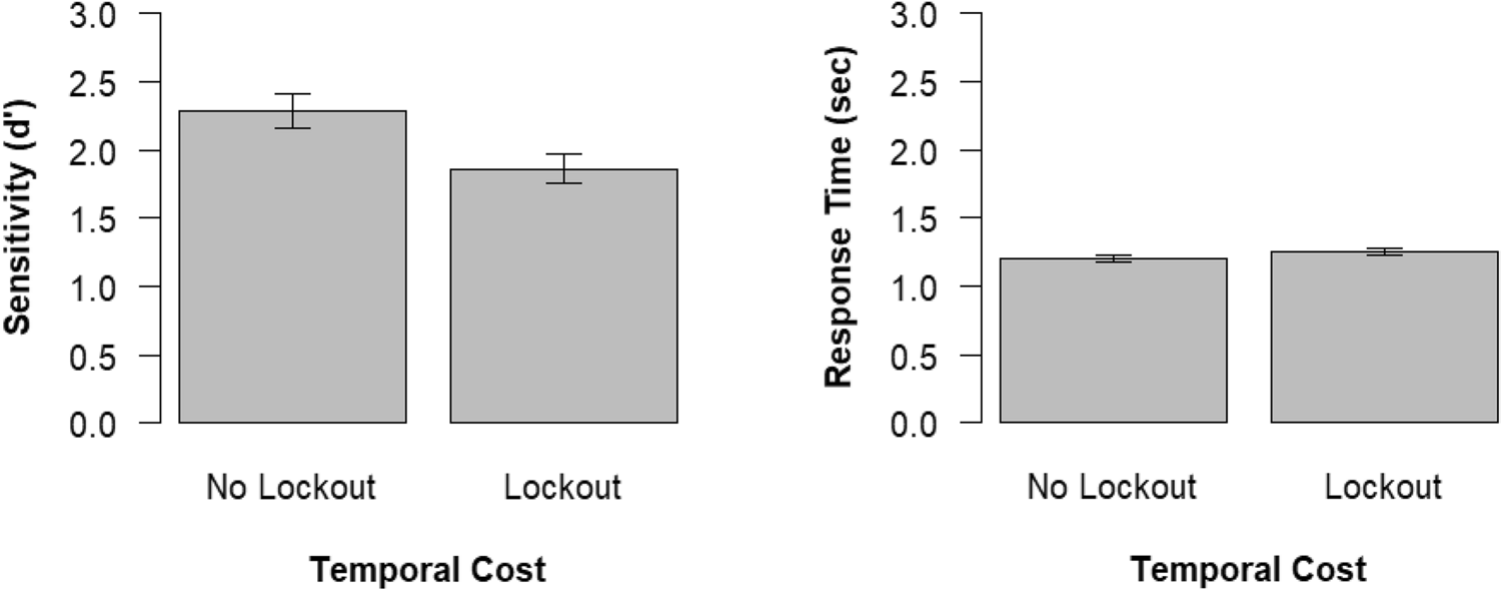
Secondary task performance (*N-*back task) in the experimental groups. Error bars indicate the standard error of the mean

We analyzed secondary task performance in the conditions in which participants responded to the *N*-back task. Following our preregistration, we conducted *t* tests for independent samples comparing sensitivity (*d′*)^10^ and response time between the condition with and without a lockout. We observed a higher sensitivity in the no lockout than lockout condition, *t*(84) = −2.60, *p* = .011, η_*p*_^2^ = .07, 95% CI [.01, .19] (see Fig. 3). Regarding response time, there was no difference between these conditions, *t*(84) = 1.27, *p* = .206, η_*p*_^2^ = .02, 95% CI [.00, .11].

### Working memory capacity

We analyzed the capacity measured by the Corsi blocks task (see Table 1) to eliminate a priori differences between the experimental groups. Following our preregistration, we conducted a 2 × 2 between-subjects ANOVA with temporal costs (no lockout vs. lockout) and secondary task (respond vs. ignore) as independent variables as well as working memory capacity as the dependent variable. There were neither significant main effects nor an interaction between the independent variables, all *F*s(1, 168) ≤ 2.98, all *p*s ≥ .086, all η_*p*_^2^s ≤ .02. For correlational analyses of working memory capacity with cognitive offloading, secondary task performance, and overall workload please consult Table S1 in the supplement. (Furthermore, exploratory ANCOVAs including working memory capacity as a covariate and offloading behavior or secondary task performance as outcome variable can be found therein.)

**Table 1:** Means and standard deviations of dependent variables in the Corsi blocks task and NASA-TLX

	No lockout		Lockout	
	Respond to secondary task	Ignore secondary task	Respond to secondary task	Ignore secondary task
	*M (SD)*	*M (SD)*	*M (SD)*	*M (SD)*
**Working memory capacity** Corsi blocks task **Overall workload**				
	4.99 (0.64) 11.63 (2.23)	4.79 (0.76) 9.12 (2.94)	4.71 (0.75) 13.16 (2.08)	4.66 (0.85) 10.82 (2.31)

### Overall workload

We exploratorily analyzed participants’ experienced workload during the experiment (as measured by the NASA-TLX). We conducted a 2 × 2 between-subjects ANOVA, with temporal costs (no lockout vs. lockout) and secondary task (respond vs. ignore) as independent variables. Participants in the lockout condition experienced the task(s) as more demanding than participants in the no lockout condition, *F*(1, 168) = 19.22, *p* < .001, η_*p*_^2^ = .10, 95% CI [.03, .19] (see Table 1). Furthermore, participants responding to the *N*-back task experienced the task performance as more demanding than participants ignoring it, *F*(1, 168) = 43.38, *p* < .001, η_*p*_^2^ = .20, 95% CI [.11, .31]. There was no significant interaction effect, *F*(1, 168) = 0.06, *p* = .809, η_*p*_^2^ < .01, 95% CI [.00, .02]. Analyses on the single variables of the NASA-TLX questionnaire (i.e., mental demand, physical demand, temporal demand, effort, frustration, performance) can be found in the supplementary material (see also Table S5).

## Discussion

We studied the interplay between offloading behavior and performance in a concurrent secondary task with the PCT in which participants were allowed to offload working memory processes and the *N*-back task capturing secondary task performance. The primary goal of our study was to investigate the impact of offloading behavior within the PCT on performance in the secondary *N*-back task. First, however, we tested how our manipulations (i.e., temporal costs and the necessity to respond to a secondary task) influenced offloading behavior. For all three indicators of offloading (i.e., openings of the model window, initial encoding duration, and initially correctly copied items), we observed that the necessity to respond to the secondary task increased offloading behavior (relative to ignoring it). Furthermore, we consistently observed a larger amount of offloading when there were no temporal costs associated with offloading (no lockout) than when offloading was associated with temporal costs (lockout). This finding replicates previous studies that showed increasing offloading behavior with decreasing temporal costs (e.g., Gray et al., 2006; Grinschgl et al., 2020).

Regarding our main offloading variable (the openings of the model window), we observed an interaction between the temporal costs and secondary task manipulations. Although responding to the secondary task increased offloading behavior in both the no lockout and lockout conditions, this increase in offloading was less pronounced when offloading was associated with temporal costs. The clearest interpretation for this interaction is that participants in the condition with temporal lockout—who ignored the secondary task—could not further reduce their offloading behavior, as memorizing more than three color locations bindings (i.e., four copy cycles) would severely challenge working memory capacity. Please note that we observed this interaction only for one of our three offloading variables. As this variable—the openings of the model window–captures offloading behavior across full trials, it might be more sensitive to our experimental manipulations, whereas the other two variables (i.e., initial encoding duration and initially correctly copied items) only capture the first opening of the model window (see Grinschgl et al., 2020, for somewhat similar differences in sensitivity).

Returning to our main research question, the pattern of secondary task performance matched that of offloading behavior. We observed more accurate secondary task performance (in terms of sensitivity) in the no lockout than in the lockout condition.^11^ Thus, an increase in offloading behavior, such as in the no lockout condition, appears to improve secondary task performance. A plausible interpretation for this finding is that cognitive offloading releases working memory resources *during* the primary task, which could be devoted to fostering the performance of a concurrent, but unrelated, secondary task. This assumption supports the flexible resource model of working memory in the context of cognitive offloading (e.g., Huang, 2010; Luck & Vogel, 2013).

Nevertheless, we should also address an alternative explanation for our finding: Possibly, the lockout condition posed an additional effort for participants due to monitoring the two-second lockout durations (e.g., checking the bar to open the model window for color changes). Thus, this increased effort of the lockout rather than the reduction in offloading behavior could have negatively affected secondary task performance in the lockout condition. In additional analyses (please see the supplement and therein Fig. S1 for details on this behalf), we tested and ruled out this possibility by excluding responses to the N-back stimuli during the lockout durations. If secondary task performance indeed would be harmed due to monitoring the lockout, excluding responses during the lockout durations (and until the model window was opened) should eliminate the observed effects. Importantly, however, the results remained unchanged; we still observed a better secondary task performance in the no lockout than the lockout condition. Thus, our primary interpretation remains likely—namely, that more offloading behavior in the no lockout condition is accompanied by released cognitive resources which are devoted to secondary task performance. We also conducted exploratory ANCOVAs (see supplement) testing whether controlling for working memory capacity would change our results—this was also not the case.

Although we observed the expected effect of offloading behavior on secondary task performance on a group-level, we did not observe correlations between offloading and secondary task performance (see Table S2 in the supplement). Furthermore, in exploratory mediation analyses (see Table S4 in the supplement), we probed whether our independent variable—the temporal costs manipulation–directly affected secondary task performance or whether this effect is mediated via cognitive offloading. Neither a direct effect of the temporal costs manipulation (no lockout vs. lockout) nor a mediated effect could be observed. Thus, on the individual level it remains unclear what drives secondary task performance. This is not unexpected as we optimized our tasks for experimental rather than correlational research (i.e., rather low variance between participants; see Hedge et al., 2018) and power for such correlational analyses was small. Therefore, we urge for studies with larger samples so that more meaningful conclusions can be drawn regarding the relationship between cognitive offloading and secondary task performance on the individual level. With regard to the group-level comparisons, however, our study shows that cognitive offloading improves secondary task performance. Nevertheless, follow-up research might extend this finding with varying study designs. For instance, it could be tested how being forced to maximally offload in the PCT (see Grinschgl et al., 2021, for such a manipulation) impacts secondary task performance in comparison to free choice offloading. Another possibility is to compare secondary task performance for participants who are not allowed to offload in the primary task and participants who are allowed to offload (note that such a study would require an easier primary task).

Analyses of the experienced workload further support the argument of cognitive offloading freeing up cognitive resources. Our participants experienced the task(s) as less demanding without the temporal lockout (i.e., more offloading) than with it (i.e., less offloading). This finding matches theoretical predictions suggesting that offloading reduces cognitive workload (Risko & Gilbert, 2016) and contradicts the idea of offloading posing additional (extraneous) load in highly demanding dual-task situations. Similarly, the participants also experienced the task(s) as less demanding when they were instructed to ignore the secondary task compared to when they had to respond to it (see Rubio et al., 2004, for converging evidence). These findings remained mostly stable when analyzing the single variables of the NASA-TLX questionnaire (i.e., mental demand, physical demand, temporal demand, effort, frustration, and performance). Please see the supplementary material and therein Table S5 for full details.

Our results reveal novel insights into the interplay between cognitive offloading and the immediate consequences of such behavior in multitasking situations. First, responding to the auditory *N*-back task elicited more offloading within the visual PCT. This suggests that handling both tasks requires the same limited working memory resources, although they address different working memory domains (auditory vs. visual working memory). This finding aligns with previous studies supporting a central capacity limitation of working memory encompassing both auditory and visual domains (e.g., Cowan, 2000; Cowan & Morey, 2007). It also stands in contrast with the competing theory of a multiple-component model of working memory (e.g., Baddeley, 2003).

Second, we observed that cognitive offloading has a beneficial impact on concurrent secondary task performance—a novel finding in the offloading literature. While previous studies highlighted that offloading improves performance in single-task setups (e.g., Arreola et al., 2019; Gilbert, 2015; Morrison & Richmond, 2020; Risko et al., 2014; Schönpflug, 1986) as well as when tasks are processed sequentially (e.g., Runge et al., 2019), our results extend these findings to highly demanding situations with multiple concurrent tasks. Combined with the work of Runge et al. (2019), who concluded that cognitive offloading releases cognitive resources that improve subsequent performance in an unrelated task, our study suggests that the performance improvements do not depend on the temporal order, but arise rather generally *during* the execution of concurrent tasks. As both of our tasks addressed different domains of working memory (visual and auditory), we speculate that these beneficial effects should cover an extensive range of unrelated tasks (at least within working memory) when encountering multitasking situations. However, it should also be further investigated how participants view the concurrent tasks (e.g., whether they are indeed perceived as separate entities or as being part of one large, complex task).

From a theoretical perspective, our study supports the notion of cognitive offloading releasing cognitive resources that can be immediately transferred to performing other tasks (Kirsh, 2010; O’Hara & Payne, 1998). This is also in line with the flexible resource model suggesting that there is a central pool of working memory resources that can be distributed across concurrent tasks if necessary (e.g., Huang, 2010; Luck & Vogel, 2013)—even across different task modalities. Thus, our study strengthens these theoretical predictions and is additionally accompanied by practical implications. Generally, performing multiple tasks concurrently results in performance deficits relative to performing the tasks separately (Appelbaum et al., 2008; Heathcote et al., 2015; Howard et al., 2020; Strobach et al., 2018). Our results suggest that these deficits—such as a high interference between concurrent tasks—could be (at least partially) compensated via cognitive offloading. Importantly, the dynamics between compensatory offloading and performance might allow participants to perform both tasks beyond the level of performance that would emerge without offloading. However, what remains unclear is to what extent cognitive offloading can compensate for the additional load when performing multiple tasks concurrently compared with performing them separately. This should be investigated in future research.

Furthermore, our study investigated cognitive offloading in a multitasking situation in which two tasks needed to be performed. When performing more than two tasks simultaneously, we would expect that offloading in at least one of the tasks should improve performance in the other tasks. However, this needs to be tested in follow-up studies. With this regard, another interesting question is whether participants would equally offload in multiple tasks if they would have the possibility to do so or whether they would rather offload in one task but not in another.

Taken together, the main messages from our study are (1) the need to perform a secondary task increased offloading behavior suggesting that both the visual pattern copy task and the auditory *N*-back task fall back onto the same working memory resources, and (2) more offloading (due to no temporal costs) in the pattern copy task improved concurrent *N*-back performance. This finding supports the flexible resource model of working memory and underlines the idea of cognitive offloading benefiting task performance even in highly demanding situations. (3) Cognitive offloading reduced participants’ perceived workload, suggesting that offloading does not pose additional effort if cognitive load is already high due to the performance of two concurrent tasks.

### Supplementary Information

ESM 1 (DOCX 190 kb)

## Data Availability

The materials and data are available at the Open Science Framework: https://osf.io/79arq/ Further, the hypothesis and analyses plan were preregistered: https://osf.io/89t24

## Footnotes

1 Throughout this article, when talking about the “primary task” we refer to a pattern copy task that allows for offloading working memory processes. When talking about the simultaneous secondary task, we refer to a *N*-back task in which offloading was prohibited. Please note, however, that the participants were not instructed that one of the tasks is more important than the other, thus, the two tasks were treated as equally important.

2 Please note that the concept of cognitive resources if often fuzzy and used to describe various different cognitive processes (Navon, 1984). When talking about cognitive resources in the present article, we specifically refer to working memory resources (i.e., unused working memory capacity).

3 This model is sometimes also referred to as *discrete-capacity model* (Souza et al., 2014).

4 It is sometimes also debated, whether the flexible resource and discrete slot models should be combined to accurately represent working memory processes (e.g., Luck & Vogel, 2013; Ma et al., 2014; Ye et al., 2017).

5 Please note that this task could also be referred to as short-term memory task (e.g., Meyerhoff et al., 2021). Often, working memory and short-term memory tasks cannot be distinguished (e.g., Colom et al., 2006; Cowan, 2008). Thus, in this paper we will also not differentiate between these constructs.

6 If participants perform at chance in the *N-*back task, their sensitivity (*d′*) would be 0 (Macmillan & Creelman, 2004). We preregistered a sensitivity of 0.5 as a cut-off value to exclude participants that did not perform the *N*-back task seriously (i.e., performing close to chance level). Two out of six participants with a sensitivity (*d′*) below 0.5 also show too large deviations (±3 *SD*) of the variables in the PCT—an additional preregistered exclusion criteria. When including the remaining four participants in our main analyses, the results do not change in a meaningful way.

7 After the lockout interval ended, the participants could open the model window as fast as they wanted. On average, they opened the model window 0.69 s after the lockout interval ended (*SD* = 0.26).

8 The auditory stimuli were retrieved from: https://de.wikipedia.org/wiki/Datei:German_alphabet-2.ogg

9 Although participants in the ignore condition were instructed to ignore the *N*-back stimuli, few participants still pressed the foot pedal on single stimuli. More specifically, 13 out of 86 participants pressed the foot pedal on some (*M* = 2.77; *SD* = 1.88) of the more than 300 stimuli. As these numbers are very low, we believe that these presses were unintended. The food pedal was placed under the desk where participants were sitting and could easily be pressed accidentally.

10 One participant achieved a hit rate of H = 1; therefore, we performed a standard correction (reducing the hit rate as if this participant made 0.5 errors) to calculate the participant’s sensitivity score (e.g., Brown & White, 2005; Stanislaw & Todorov, 1999).

11 Please note that we did not observe significant group differences with regard to response time in the *N*-back task. The design of the N-back task has likely encouraged participants to respond accurately instead of focusing on speed.
