# Supplementary material for: Mutual interplay between cognitive offloading and secondary task performance
Source: Psychon Bull Rev. 2023 Jun 13;30(6):2250–61. doi: 10.3758/s13423-023-02312-3 (PMC10728259; doi:10.3758/s13423-023-02312-3)
Supplement: Supplementary file 1 — (DOCX 190 kb) [file 13423_2023_2312_MOESM1_ESM.docx]

Supplementary Material

**Additional Analyses for Secondary Task Performance**

Within our main analyses, we observed a more accurate secondary task performance for participants in the no lockout condition than in the lockout condition. We argue that this effect arises from the increase in offloading behavior in the no lockout condition which comes along with an increasing amount of released resources that could be directed towards the performance in the secondary task. However, another explanation might be that participants in the lockout condition experience more effort as they might have to monitor the two-second lockout durations and thus show a reduced secondary task performance. To rule out this alternative explanation, we conducted exploratory analyses for the secondary task performance. More specifically, we excluded responses to stimuli in the N-back Task which coincided with the lockout durations (including the individual time intervals for opening the model window after the 2 s lockout) of each trial. If monitoring the lockout durations poses a higher effort and thus reduces secondary task performance, our observed effect should diminish when responses during the lockout durations are excluded from the analyses.

Using *t-*Tests, we compared secondary task performance between the no lockout condition and the new scores in the lockout condition (i.e. without responses to stimuli that were presented or responded to during the active lockout and until participants opened the model window).^[[1]](#footnote-1)^ As in our main analyses, we observed a higher sensitivity (d’) in the no lockout than the lockout condition, *t*(83) = −2.59, *p* = .011, η*_p_*^2^ = .07, 95% CI [.003; .19] (see Figure S1). For response time, we did not observe any group differences, *t*(83) = 1.57, *p*= .119, η*_p_*^2^ = .03, 95% CI [.00; .13].

To conclude, even when responses to stimuli that were played during the lockout or to which participants reacted during the lockout (and until the model window was opened) are excluded, the lockout group showed a worse secondary task performance (in terms of sensitivity) than the no lockout group. Thus, it appears that a potentially higher effort due to monitoring the lockout is not the reason for the reduced secondary task performance in the lockout condition. Instead, less offloading in this condition in comparison to the no lockout condition likely causes harms for secondary task performance.

**Figure S1.** Secondary task performance (N-back Task) in the experimental groups. In the lockout group, responses to stimuli that were played during the lockout durations or to which participants reacted during the lockout durations were excluded. Error bars indicate the standard error of the mean.


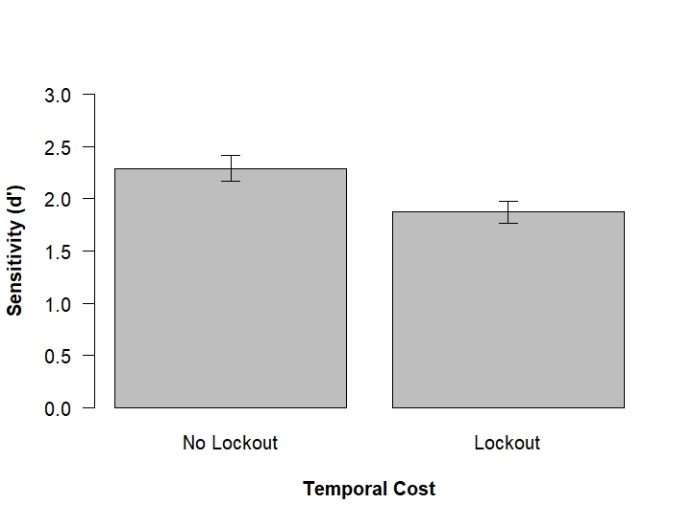

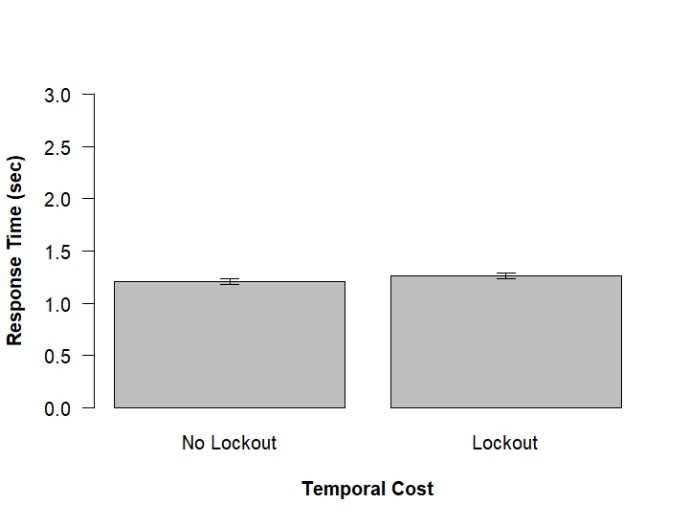


**Correlations**

Table S1

*Correlations of working memory capacity with cognitive offloading, secondary task performance, and overall workload*

|  | **Working Memory Capacity** | | | |
| --- | --- | --- | --- | --- |
|  | No lockout | | Lockout | |
|  | Respond to secondary task | Ignore secondary task | Respond to secondary task | Ignore secondary task |
| **Cognitive Offloading** |  | | | |
| Openings of the Model Window | -.11 | -.54*** | -.18 | -.43** |
| Initial Encoding Duration (sec) | -.17 | -.01 | -.14 | .29 |
| Initially Correctly Copied Items | .02 | .34* | .03 | .37* |
| **Secondary Task Performance** |  | | | |
| Sensitivity (d’) | .29 | - | .32* | - |
| Response Time (sec) | -.32* | - | .16 | - |
| **Overall Workload** | -.08 | -.06 | -.02 | .15 |

*Note:* *N (*per group) = 43; * *p* < .05, ** *p* < .01, *** *p* < .001

Table S2

*Correlations of cognitive offloading and secondary task performance*

|  | **Openings of the Model Window** | |
| --- | --- | --- |
|  | No lockout | Lockout |
| **Sensitivity (d’)**  **Response Time (sec)** | .02  -.06 | .01  -.11 |
|  | **Initial Encoding Duration (sec)** | |
|  | No lockout | Lockout |
| **Sensitivity (d’)**  **Response Time (sec)** | -.21  -.01 | -.04  .06 |
|  | **Initially Correctly Copied Items** | |
|  | No lockout | Lockout |
| **Sensitivity (d’)**  **Response Time (sec)** | <.01  .04 | -.14  .03 |

*Note:* *n (*per group) = 43; * *p* < .05, ** *p* < .01, *** *p* < .001

Table S3

Correlation of overall workload with cognitive offloading and secondary task performance

|  | **Overall Workload** | | | |
| --- | --- | --- | --- | --- |
|  | No lockout | | Lockout | |
|  | Respond to secondary task | Ignore secondary task | Respond to secondary task | Ignore secondary task |
| **Cognitive Offloading** |  | | | |
| Openings of the Model Window | .01 | .02 | -.05 | .11 |
| Initial Encoding Duration (sec) | .28 | .41** | .02 | .17 |
| Initially Correctly Copied Items | .04 | -.03 | -.04 | -.17 |
| **Secondary Task Performance** |  | | | |
| Sensitivity (d’) | .03 | - | -.09 | - |
| Response Time (sec) | -.16 | - | .13 | - |

*Note:* *n (*per group) = 43; * *p* < .05, ** *p* < .01, *** *p* < .001

**ANCOVAs with working memory capacity as a covariate**

To test whether our findings for offloading behavior and secondary task performance remain the same when controlling for working memory capacity as a covariate, we conducted exploratory ANCOVAs.

For offloading behavior, the covariate “working memory capacity” was significantly related to the number of openings of the model window as well as the initially correctly copied items, all *F*(1, 167)s ≥ 9.07, all *p*s < .003, whereas it was not related to the initial encoding duration, *F*(1, 167) = 0.88, *p* = .349. Most importantly, as in our original analyses we observed significant main effects of the temporal costs, all *F*(1, 167)s ≥ 41.08, all *p*s < .001, and the necessity to respond to the secondary task manipulations, all *F*(1, 167)s ≥ 10.62, all *p*s < .001, across all three indicators of offloading. Furthermore, for one variable–the number of openings of the model window–we observed an interaction effect reflecting our original analyses, *F*(1, 167) = 18.65, *p* < .001, whereas no interaction effect for the other two offloading variables was found, all *F*(1, 167)s ≤ 3.10, all *p*s ≥ .080.

With regard to secondary task performance, working memory capacity as a covariate was related to sensitivity (d’), *F*(1, 83) = 8.79, *p* = .003, but not to response time, *F*(1, 83) = 0.37, *p* = .546. In line with our original analyses, for sensitivity (d’) we observed a significant difference between the no lockout and lockout condition even when controlling for working memory capacity, *F*(1, 83) = 4.31, *p* = .041. For response time, no such difference was observed, *F*(1, 83) = 1.26, *p* = .264. To conclude, considering working memory capacity as a covariate in our analyses does not change our results and interpretations.

**Mediation Analyses**

Table S4

|  | **Secondary Task Performance** | | | | | | | |
| --- | --- | --- | --- | --- | --- | --- | --- | --- |
|  | Sensitivity (d‘) | | | | | | | |
| **Cognitive Offloading** | Mediated effect | 95% CI | Direct effect | 95% CI | Total Effect | 95% CI | Prop. Mediated | 95% CI |
| Openings of the Model Window | -0.03 | [-0.39; 0.39] | -0.40 | [-0.94; 0.14] | -0.43** | [-0.75; -0.12] | 0.07 | [-0.41; 4.63] |
| Initial Encoding Duration (sec) | -0.11 | [-0.35; 0.12] | -0.31 | [-0.76; 0.13] | -0.43** | [-0.75; -0.12] | 0.26 | [-1.62; 0.56] |
| Initially Correctly Copied Items | -0.07 | [-0.28; 0.19] | -0.36 | [-0.80; 0.06] | -0.43** | [-0.75; -0.12] | 0.16 | [-0.35; 2.02] |
|  | Response Time (sec) | | | | | | | |
| Openings of the Model Window | 0.03 | [-0.07; 0.09] | 0.02 | [-0.07; 0.12] | 0.05 | [-0.03; 0.12] | 0.56 | [-6.42; 6.27] |
| Initial Encoding Duration (sec) | 0.01 | [-0.04; 0.06] | 0.03 | [-0.05; 0.13] | 0.05 | [-0.03; 0.12] | 0.28 | [-4.01; 5.48] |
| Initially Correctly Copied Items | <0.01 | [-0.06; 0.04] | 0.04 | [-0.04; 0.12] | 0.05 | [-0.03; 0.12] | 0.04 | [-2.51; 5.37] |

*Mediation analyses with the predictor lockout (no lockout vs. lockout), mediator cognitive offloading, and criterion secondary task performance*

*Note:* *n* = 86; * *p* < .05, ** *p* < .01, *** *p* < .001; all mediation analyses were conducted with a bootstrapping procedure (1000 simulations) using the package “mediation” in R (Tingley et al., 2014)

**Additional Analyses for the NASA-TLX questionnaire**

Besides analyzing overall workload (see main manuscript), we also exploratorily tested effects of our temporal costs (no lockout vs. lockout) and secondary task manipulations (respond vs. ignore) on the single variables of the NASA-TLX questionnaire (mental demand, physical demand, temporal demand, effort, frustration, performance). Using 2 x 2 between-subjects ANOVAs, we observed that participants in the lockout condition experienced the task(s) as more mentally demanding, experienced a higher effort, more frustration, and a worse performance than participants in the no lockout condition, all *F*(1, 168)s ≥ 5.15, all *p*s ≤ .024, all η*_p_*^2^s ≥ .03 (see Table S5). Furthermore, participants responding to the N-back Task experienced the tasks as more mentally demanding, experienced a higher effort, more frustration, and a worse performance than participants ignoring this task, all *F*(1, 168)s ≥ 4.78, all *p*s ≤ .030, all η*_p_*^2^s ≥ .02. For physical and temporal demand, we observed a main effect of the necessity to perform the secondary task. Participants that responded to the secondary task showed a higher physical and temporal demand than participants not responding to it, all *F*(1, 168)s ≥ 9.19, all *p*s ≤ .003, all η*_p_*^2^s ≥ .09. No main effect of the temporal costs manipulation was observed for physical and temporal demand, all *F*(1, 168)s ≤ 3.01, all *p*s ≥ .081, all η*_p_*^2^s ≤ .02. For all variables, no interaction effects were significant, all *F*(1, 168)s ≤ 1.08, all *p*s ≥ .299, all η*_p_*^2^s ≤ .01.

Table S5

*Means and Standard Deviations of Dependent Variables in the NASA-TLX*

|  | No lockout | | Lockout | | |
| --- | --- | --- | --- | --- | --- |
|  | Respond to secondary task | Ignore secondary task | Respond to secondary task | Ignore secondary task | |
|  | *M (SD)* | *M (SD)* | *M (SD)* | | *M (SD)* |
| Overall workload  Mental demand  Physical demand  Temporal demand  Effort  Frustration  Performance | 11.63 (2.23)  15.40 (3.01)  5.53 (3.77)  12.88 (3.78)  15.23 (3.63)  9.35 (4.98)  11.40 (3.92) | 9.12 (2.94)  11.16 (4.43)  4.26 (3.75)  10.30 (3.53)  12.51 (4.79)  7.28 (4.96)  9.21 (3.76) | 13.16 (2.08)  17.72 (2.29)  6.81 (4.45)  11.72 (3.93)  16.65 (2.49)  12.88 (5.38)  13.14 (4.32) | | 10.82 (2.31)  14.28 (3.39)  4.42 (3.89)  9.42 (4.03)  15.07 (3.03)  11.47 (5.58)  10.28 (4.23) |

1. In line with our preregistered exclusion criteria, for this analysis one participant was excluded from the lockout group due to a sensitivity (d’) lower than 0.5. Including this participant does not change the results in a meaningful way. [↑](#footnote-ref-1)
